# *Limosilactobacillus fermentum* ANC4 (KCTC 15072BP) Mitigates Dexamethasone-Induced Muscle Atrophy and Improves Overall Skeletal Muscle Function

**DOI:** 10.4014/jmb.2603.03025

**Published:** 2026-04-28

**Authors:** Seong-Min Hong, Jinho Park, Su-Hyun Kim, Ahyoung Hwang, Eun Sung Jung, Donghyun Cho, Yosep Ji, Sun Yeou Kim, Choong-Hwan Lee

**Affiliations:** 1 College of Pharmacy and Gachon Institute of Pharmaceutical Sciences, Gachon University, Incheon 21936, Republic of Korea; 2 Division of Food Science and Technology and Institute of Agriculture and Life Science, Gyeongsang National University, Jinju, Gyeongnam 52828, Republic of Korea; 3 Department of Sports Rehabilitation, Gimcheon University, Gimcheon 39528, Republic of Korea; 4 Department of Bioscience and Biotechnology, Konkuk University, Seoul 05029, Republic of Korea; 5 HEM Pharma Inc., Suwon 16229, Republic of Korea

**Keywords:** *Limosilactobacillus fermentum* ANC4, Muscle atrophy, Dexamethasone, Gastrocnemius, Myogenic regeneration, Probiotic, Postbiotic

## Abstract

Glucocorticoid-induced muscle atrophy involves accelerated protein breakdown and impaired regeneration. We evaluated the protective effects of heat-killed and live *Limosilactobacillus fermentum* ANC4 against dexamethasone (Dex)-induced muscle wasting in C57BL/6 mice. Oral administration of *L. fermentum* ANC4 significantly improved grip strength and treadmill endurance while specifically preserving the mass of the gastrocnemius (GCM), a fast-twitch-dominant muscle. Mechanistically, *L. fermentum* ANC4 downregulated the glucocorticoid receptor (GR) and E3 ubiquitin ligases, including muscle RING-finger protein-1 (MuRF-1) and Atrogin-1, thereby mitigating catabolic signaling. Simultaneously, it up-regulated myogenic regenerative markers, including myogenic differentiation 1 (MyoD), myogenin, and myosin heavy chain (MyHC). Histological analysis confirmed that muscle fiber size restored and reduced fibrosis. Notably, the heat-killed form effectively increased MyHC, while the live form strongly suppressed Atrogin-1. These findings suggest that *L. fermentum* ANC4 prevents muscle atrophy by balancing catabolic signaling and myogenic repair, highlighting its potential as a probiotic or postbiotic therapy for preserving muscle mass and function.

## Introduction

Muscle atrophy results in progressive loss of skeletal muscle mass and strength. While muscle loss is a hallmark of various conditions such as aging-related sarcopenia and cancer-induced cachexia, the present study specifically focuses on glucocorticoid-induced muscle atrophy (GIA) [1-3]. GIA is a serious clinical complication resulting from the long-term or high-dose use of synthetic steroids like dexamethasone (Dex), which are widely prescribed for chronic inflammatory and autoimmune diseases [4]. This pharmacological form of muscle wasting significantly impairs physical mobility and quality of life, often limiting the clinical efficacy of essential steroid treatments.

Biologically, atrophy occurs when protein degradation outpaces synthesis. This shift typically involves catabolic pathways that speed up proteolysis [5, 6]. Among these, the ubiquitin-proteasome system (UPS) is a key regulator of muscle atrophy, with transcriptional activation of E3 ubiquitin ligases, such as muscle RING finger-1 (MuRF-1) and muscle atrophy F-box (Atrogin-1) being central to muscle protein breakdown [7]. While our understanding of the molecular drivers of muscle wasting has significantly improved, the development of safe and effective therapies to prevent or reverse muscle atrophy remains a major challenge.

Recently, probiotics have emerged as a potential solution owing to their ability to influence metabolism and inflammation. Probiotics are live microorganisms that provide health benefits when ingested in sufficient amounts [8-10]. For instance, strains from the *Lactobacillus* and *Bifidobacterium* families are known to reduce inflammation and oxidative stress in various disease models [9-11]. Probiotics and their metabolites, including short-chain fatty acids (SCFAs), branched-chain amino acids (BCAAs), and other bioactive compounds (*e.g.*, Conjugated linoleic acid, Urolithin A), have been reported to modulate muscle homeostasis *via* the gut-muscle axis [11-14]. For instance, certain *Lactobacillus* and *Limosilactobacillus* strains have been shown to regulate muscle protein synthesis and degradation pathways in dexamethasone-induced atrophy models, potentially through the systemic circulation of these diverse microbial products [15, 16].

While robust survivability within the gastrointestinal tract is a fundamental prerequisite for probiotics to exert systemic effects, *Limosilactobacillus fermentum* is a particularly strong candidate for mitigating muscle atrophy due to its proven antioxidant capacity and ability to modulate systemic inflammation [17, 18]. These functional properties are essential for counteracting the oxidative stress and catabolic signaling pathways associated with dexamethasone-induced muscle wasting [19]. In this study, we selected *L. fermentum* ANC4 (KCTC 15072BP), a strain isolated from makgeolli (Korean rice wine), for its confirmed safety profile and superior functional properties including high gastrointestinal stability, potent antioxidant capacity, and protective effects against glycotoxicity demonstrated in our preliminary screenings. These validated traits make ANC4 a promising candidate for counteracting oxidative stress and inflammation associated with skeletal muscle atrophy. However, its specific effects on skeletal muscle protection remain incompletely explored.

Increasing evidence indicates the “gut–muscle axis” as a two-way communication channel that involves metabolites, immune signals, and hormones as a critical factor for muscle health [20]. In this study, the gut-muscle axis served as the conceptual rationale for selecting a probiotic strain. While we focused on the downstream effects within the skeletal muscle, we hypothesized that oral administration of *L. fermentum* ANC4 would attenuate muscle atrophy by suppressing catabolic signaling and enhancing myogenic pathways. We thus propose that *L. fermentum* ANC4 may offer a protective shield against muscle atrophy, potentially by recalibrating the balance between protein degradation and regeneration. To investigate this in detail, we subjected mice to Dex-induced muscle atrophy and treated them with live or heat-killed *L. fermentum* ANC4. Subsequent analysis, including functional tests, structural examination, and molecular profiling, was designed to verify the potential utility of the strain as a novel probiotic or postbiotic therapy.

## Materials and Methods

### Preparation of *L. fermentum* ANC4

We cultured *L. fermentum* ANC4 (KCTC15072BP) under aerobic conditions at 37°C, using de Man, Rogosa, and Sharpe (MRS) broth containing 0.5% L-cysteine. For the seed culture, we inoculated a single colony into 5 mL of MRS broth and incubated it for 18 h. This pre-culture was then adjusted to an optical density (OD_600_) of 1.0 and inoculated at 1% (v/v) into 1 L of fresh sterile MRS broth, followed by a 24 h incubation at 37°C. To prepare heat-killed bacteria, we resuspended the harvested cells in phosphate-buffered saline (PBS) at 2 × 10^10^ CFU/mL and heated them in a water bath at 95°C for 15 min. For the live preparation, the cells were harvested by centrifugation and resuspended in sterile PBS. Both preparations were diluted to the required dosages (5 × 10^9^ CFU/mL and 2 × 10^10^ CFU/mL) and stored at 4°C until administration [21, 22].

### Animal Study Design

**Fig. 1:**
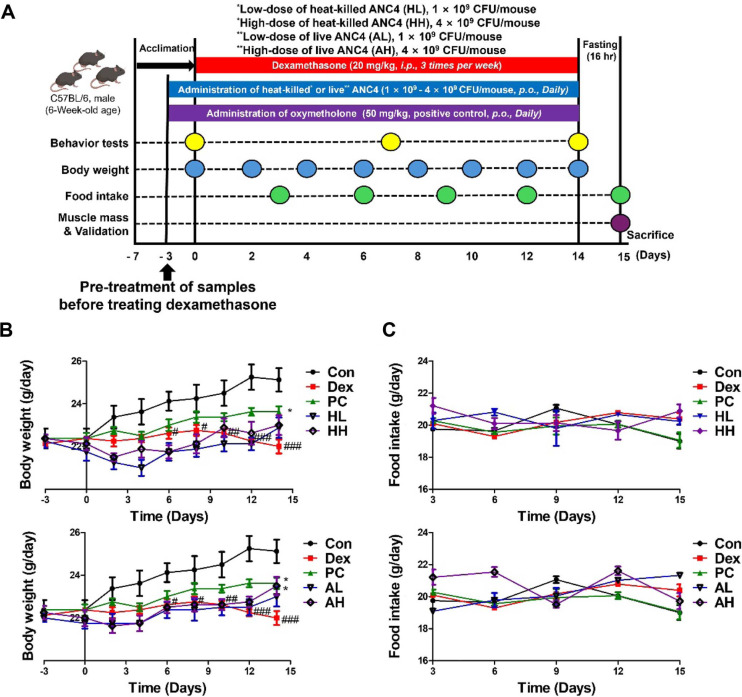
Experimental design and effects on body weight and food intake. (**A**) Schematic representation of the in vivo experimental procedure. After acclimation, mice were randomly assigned to seven groups (n = 8 per group): Control (Con), dexamethasone-induced (Dex, 20 mg/kg), low-dose heat-killed *L. fermentum* ANC4 (HL, 1 × 10^9^ CFU/mouse), high-dose heat-killed *L. fermentum* ANC4 (HH, 4 × 10^9^ CFU/mouse), low-dose live *L. fermentum* ANC4 (AL, 1 × 10^9^ CFU/mouse), high-dose live *L. fermentum* ANC4 (AH, 4 × 10^9^ CFU/mouse), and positive control with oxymetholone (PC, 50 mg/kg). (B, C) Body weight and food intake were monitored weekly throughout the study period. In panels B and C, the upper graphs compare the Con, Dex, and PC groups to establish the muscle atrophy model and positive control efficacy, while the lower graphs compare the Dex group with the four treatment groups (HL, HH, AL, and AH) to evaluate the dose-dependent effects of live and heat-killed *L. fermentum* ANC4. ^#^*p* < 0.05, ^##^*P* < 0.01, and ^###^*p* < 0.001 vs. control (Con) group; *p < 0.05 vs. Dex-induced group (Dex) (Two-way ANOVA test). All data represent the mean ± SEM (n = 8 per group).

We obtained six-week-old male C57BL/6 mice, allowed a one-week acclimation period, and randomly divided them into seven groups (n = 8 per group; Fig. 1A) as follows: (1) Control (Con), (2) Dexamethasone only (Dex, 20 mg/kg), (3) Positive Control (PC, Dex + 50 mg/kg oxymetholone), (4) Dex + Low-dose Heat-killed *L. fermentum* ANC4 (HL, 1 × 10^9^ CFU), (5) Dex + High-dose Heat-killed *L. fermentum* ANC4 (HH, 4 × 10^9^ CFU), (6) Dex + Low-dose Live *L. fermentum* ANC4 (AL, 1 × 10^9^ CFU), and (7) Dex + High-dose Live *L. fermentum* ANC4 (AH, 4 × 10^9^ CFU). To induce muscle atrophy, we injected Dex intraperitoneally (20 mg/kg) three times a week for two weeks. We administered probiotic treatments (orally in PBS) daily, starting three days before the first Dex injection, and continued until the end of the study. Oxymetholone (50 mg/kg per day) was administered as a positive control. Oxymetholone (50 mg/kg per day, PC) served as a positive control due to its established anabolic efficacy in glucocorticoid induced atrophy models [23, 24]. This dose was selected to ensure a robust comparison of the anti-atrophic potential of the tested preparations. The study protocol was reviewed and approved by the Institutional Animal Care and Use Committee of Gachon University (Approval No. GU1-2023-IA0032).

### Assessment of Physical Function

To evaluate muscle function, we conducted treadmill running and grip strength tests at baseline and on days 7 and 14. For grip strength assessment, we used a grip strength meter (JD-A-22; Jungdo B&P, Republic of Korea) [25]. Each mouse was placed on a horizontal metal grid and gently pulled backward by its tail until it released its grip. We recorded the peak tension (g) three times per mouse and used the average value for the analysis. We used a motorized treadmill (JD-A-22) for endurance testing. After an adaptation week, the mice ran for 30 min: a 5 min warm-up at 5 m/min, followed by 20 min of acceleration (5–20 m/min), and a 5 min cool-down at 10 m/min. The total running time and average speed were recorded weekly.

### Muscle Tissue Collection

At the end of the 14-day period, the mice were sacrificed and the hindlimb muscles, including the quadriceps femoris (QD), gastrocnemius (GCM), soleus (SOL), plantaris (PLA), and extensor digitorum longus (EDL), were dissected. The collected muscles were briefly rinsed in ice-cold PBS to remove residual blood and surface fat. The wet tissues were immediately weighed using a precision balance, and muscle weights were normalized to the total body weight (mg/g) to account for variations in animal size.

### Western Blot Analysis

We extracted proteins from the GCM tissues by homogenizing them in ice-cold RIPA buffer containing protease and phosphatase inhibitors. After centrifuging the lysates at 12,000 × g for 1 h at 4°C, we collected the supernatants and measured the protein concentrations using a Bradford assay. Equal amounts of protein (30–40 μg) were separated on SDS-PAGE gels and transferred onto PVDF membranes. Following a 1 h blocking step with 5% BSA in TBS-T, we incubated the membranes overnight at 4°C with primary antibodies against glucocorticoid receptor (GR, 1:1000, Abcam, UK), MuRF-1 (1:1000, Abcam), Atrogin-1 (1:1000, Abcam), Myogenin (1:1000, Abcam), MyoD (1:1000, Abcam), myoblast determination protein and myosin heavy chain (MyHC, 1:1000, Santa Cruz Biotechnology, USA), and α-tubulin (clone 236-10501, Invitrogen, USA). After washing, the membranes were incubated with horseradish peroxidase-conjugated secondary antibodies (1:2000) for 1 h at room temperature. Protein bands were visualized using enhanced chemiluminescence (ECL) reagents and quantified using Image Lab software (Bio-Rad, USA).

### Histological Analysis

**Hematoxylin and eosin (H&E) staining.** We fixed the GCM, liver, and kidney samples in 10% neutral buffered formalin for 24 hours, embedded them in paraffin, and cut them into 5-μm sections. The sections were then stained with hematoxylin and eosin (H&E) to observe general tissue architecture. Images were captured using a light microscope (Olympus, Japan) at 20× magnification. The cross-sectional area (CSA) of the muscle fibers was quantified using ImageJ software (version 1.53, NIH, USA). To ensure representative sampling and reproducibility, five randomly selected fields per slide were evaluated (n = 3 to 4 per group), from at least 100 randomly selected fibers per sample.

**Masson's trichrome staining.** To evaluate fibrosis, GCM sections were stained with Masson trichrome according to the manufacturer instructions (Abcam). Collagen fibers were stained blue and muscle fibers were stained red. For quantitative analysis, the Masson positive area was calculated using ImageJ software (version 1.53, NIH). Blue stained collagenous regions were isolated from the background using the Color Deconvolution plugin specifically calibrated for the Masson trichrome vector. A standardized threshold range within the RGB color space was established and applied uniformly across all histological sections to define the positive area, ensuring the reproducibility of the analysis. The quantification was performed in five randomly selected fields per slide (n = 3 to 4 per group). The fibrotic area is expressed as a percentage of the total tissue area. To minimize investigator bias, the entire quantification process was performed in a blinded manner by a trained researcher.

**Immunohistochemistry (IHC) staining.** The GCM sections were deparaffinized, and antigen retrieval was performed in citrate buffer. After blocking endogenous peroxidase and non-specific binding, we incubated the slides overnight with an antibody against MyoD (1:200; Abcam). We then applied a biotinylated secondary antibody and an avidin-biotin-peroxidase complex (Vector Laboratories). Signals were developed using a DAB substrate, and nuclei were counterstained with hematoxylin. Images were obtained using a light microscope (Olympus) and the optical density of MyoD staining was analyzed using ImageJ software (version 1.53, NIH). Quantitative assessment was conducted in five randomly selected fields per slide (n = 3 to 4 per group) to ensure the accuracy and representativeness of the results.

### Statistical Analysis

All results were expressed as the mean ± standard error of the mean (SEM). One-way analysis of variance (ANOVA) followed by Tukey’s post-hoc test was used to compare differences between groups. For data measured over time (body weight, grip strength, and running performance), we applied a two-way repeated-measures ANOVA. We considered a *p*-value less than 0.05 to be statistically significant and performed all analyses using GraphPad Prism 5.0 software.

## Results

### *L. fermentum* ANC4 Protects Against Dex-Induced Weight Loss Without Altering Food Consumption

As specified in Figure 1B, the baseline body weight was comparable across all experimental groups at day -3. Over the 14-day injection period, the Dex-treated group experienced severe stagnation in weight gain compared with the healthy control group (^###^*P* < 0.001, Fig. 1B). Interestingly, during the initial phase from day 0 to day 8, the HL, HH, AL, and AH groups showed a transient reduction in body weight. This initial dip is attributed to the cumulative stress of daily oral handling and the physiological adaptation of the intestinal environment to probiotic administration. However, following this adaptation period, oral administration of *L. fermentum* ANC4 effectively counteracted the weight loss in a dose dependent manner. Notably, the high dose live *L. fermentum* ANC4 (AH) group exhibited a statistically significant recovery in body weight that was comparable to that of the positive control group treated with oxymetholone (50 mg/kg, PC). Daily food intake did not differ significantly among any of the groups throughout the study (Fig. 1C). These results demonstrated that *L. fermentum* ANC4 alleviates dexamethasone induced weight loss independently of food intake regulation and without inducing acute toxicity.

### *L. fermentum* ANC4 Improves Grip Strength and Treadmill Performance in Dex-Induced Muscle Atrophy

**Fig. 2:**
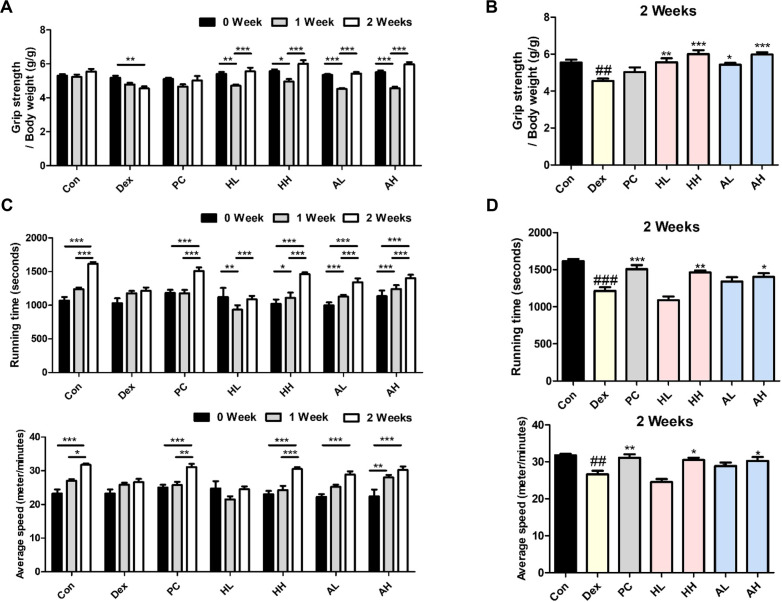
Effects of *L. fermentum* ANC4 on grip strength and treadmill exercise performance. (**A**) Grip strength tests were performed in each group to evaluate the effect of muscular strength on Dex-induced atrophic alterations after each weekly treadmill exercise. *p < 0.05, **p < 0.01, and ***P < 0.001 vs. 0 week (Twoway ANOVA test). (**B**) The final grip strength was assessed in the last week of the experiment. ^##^*P* < 0.01 vs. control group (Con). *p < 0.05, **p < 0.01, and ***p < 0.001 vs. Dex-induced group (Dex). (**C**) For comparison within groups, the running time (s) and average speed (m/min) were recorded after each weekly treadmill exercise. *p < 0.05 and ***p < 0.001 vs. 0 week (Two-way ANOVA test). (**D**) The final treadmill endurance (running time and average speed) was measured in the last week. ^##^*p* < 0.01 and ^###^*p* < 0.001 vs. control group (Con). *p < 0.05, **p < 0.01, and ***p < 0.001 vs. Dex-induced group (Dex). All data represent the mean ± SEM (n = 8 per group).

We then evaluated the effects of *L. fermentum* ANC4 on muscle function using grip strength and treadmill endurance tests. In the grip strength assessment, the Dex-induced group showed a significant decline in muscle strength (4.55 ± 0.13 g/g) compared with that in the control group (5.55 ± 0.16 g/g; ^##^*p* < 0.01) at the final test point (Fig. 2A). However, treatment with *L. fermentum* ANC4 effectively reversed this deficit. Both the high-dose heat-killed (HH) and live (AH) groups showed significant recovery starting from week 1. By week 2, the HH and AH groups achieved grip strength values of 6.00 ± 0.22 g/g and 5.98 ± 0.12 g/g, respectively. These values were significantly higher than those of the Dex group (***p < 0.001) and surpassed the positive control (PC) group (5.03 ± 0.26 g/g) as shown in Fig. 2B.

Regarding treadmill endurance, Dex administration markedly suppressed physical performance, resulting in reduced running time and average speed compared with those of the control group. However, treatment with high-dose *L. fermentum* ANC4 significantly restored endurance capacity (***p < 0.001; Fig. 2C). Specifically, the HH group recorded a running time of 1462.63 ± 26.99 s and an average speed of 30.50 ± 0.63 m/min. Similarly, the AH group showed a running time of 1401.75 ± 50.07 s and an average speed of 30.25 ± 1.05 m/min. These functional improvements were comparable to the performance of the PC group, which exhibited a running time of 1507.75 ± 55.13 s and an average speed of 31.13 ± 0.93 m/min (See Fig. 2D). Overall, *L. fermentum* ANC4, particularly in its high-dose form, effectively mitigated Dex-induced functional impairment, restoring both muscle strength and endurance to near-normal levels.

### *L. fermentum* ANC4 Increases Skeletal Muscle Mass

**Fig. 3:**
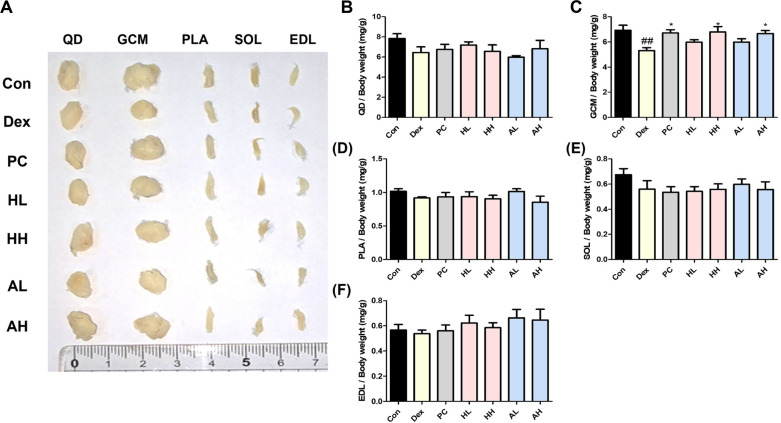
Effects of *L. fermentum* ANC4 on lower limb skeletal muscle mass. (**A**) Representative images of dissected wet muscles, including quadriceps femoris (QD), gastrocnemius (GCM), soleus (SOL), plantaris (PLA), and extensor digitorum longus (EDL). (**B–F**) Quantitative analysis of wet muscle weights from the right hindlimb. ^##^*p* < 0.01 vs. control group (Con). *p < 0.05 vs. Dex-induced group (Dex). All data represent the mean ± SEM (n = 8 per group).

The muscles of the lower limb, specifically the QD, GCM, SOL, PLA, and EDL are fundamental for maintaining posture and executing motor movements [26]. Compared with the control group, the Dex-induced group showed a tendency toward decreased mass in these muscles. However, a statistically significant reduction was specifically observed in the GCM muscle compared to the control group. We recorded wet weights of 6.45 ± 0.48 mg/g, 6.46 ± 0.48 mg/g, 0.92 ± 0.01 mg/g, 0.56 ± 0.06 mg/g, and 0.54 ± 0.03 mg/g for the QD, GCM, SOL, PLA, and EDL (Fig. 3). While oral administration of *L. fermentum* ANC4 showed a restorative trend across various muscle types, its protective effect reached statistical significance notably in the GCM. (Fig. 3A and 3C). In particular, the high-dose groups demonstrated robust protection, with the weight of the GCM in the heat-killed (HH) group reaching 6.56 ± 0.56 mg/g and that in the live (AH) group reaching 6.83 ± 0.72 mg/g (*p < 0.05). These values were on par with that obtained in the positive control group (6.75 ± 0.51 mg/g, *p < 0.05) (Fig. 3C). These results, including representative images of muscle morphology (Fig. 3A) and quantified GCM muscle mass (Fig. 3C), support previous findings that the GCM is particularly sensitive to atrophic stimuli. Given that Dex-induced atrophy primarily targets fast-twitch (Type II) fibers, the significant preservation of the GCM as a muscle with an overwhelmingly high proportion of fast-type fibers highlights the targeted efficacy of the *L. fermentum* ANC4 strain in mitigating glucocorticoid-induced muscle loss [5]. Therefore, *L. fermentum* ANC4 may serve as a promising anti-atrophic agent for preserving or improving muscle mass, particularly in fast-twitch muscles such as the GCM.

### *L. fermentum* ANC4 Modulates the Expression of Muscle Atrophy and Regeneration Markers

**Fig. 4:**
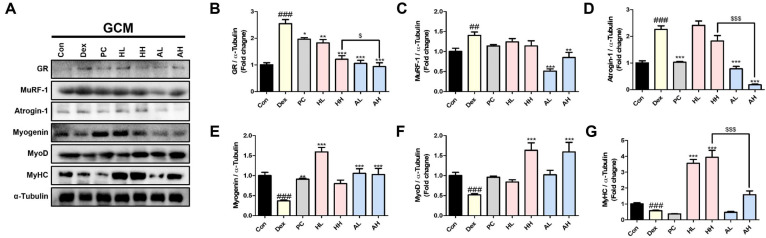
Effects of *L. fermentum* ANC4 on atrophy-related protein expression in the gastrocnemius (GCM) muscle. (**A**) Representative western blot images of GR, MuRF-1, Atrogin-1, Myogenin, MyoD, and MyHC protein levels. α-Tubulin was used as the loading control. (**B–G**) Quantitative analysis of protein band intensities using Bio-Rad Quantity One software. ^##^*p* < 0.01 and ^###^*p* < 0.001 vs. control group (Con). *p < 0.05, **p < 0.01, and ***p < 0.001 vs. Dex-induced group (Dex). ^$^*p* < 0.05 and ^$$$^*p* < 0.001 vs. High-dose Heat-killed *L. fermentum* ANC4 (HH) group. All data represent the mean ± SEM (n = 8 per group).

As the GCM muscle is highly sensitive to atrophic stimuli, we focused our molecular analysis on this tissue to determine the mechanisms underlying muscle wasting and repair. The glucocorticoid receptor (GR) is a central driver of atrophy, as it directly triggers the transcription of catabolic genes, such as MuRF-1 and Atrogin-1 [27]. Western blot analysis revealed that Dex administration increased GR expression by approximately threefold compared with that in the control group (^###^*p* < 0.001) (Fig. 4A and 4B). This activation of glucocorticoid signaling was accompanied by a distinct increase in downstream E3 ubiquitin ligases. Specifically, MuRF-1 levels increased 1.4-fold (^##^*p* < 0.01) and Atrogin-1 increased roughly 2.3-fold (^###^*p* < 0.001), confirming a shift toward a catabolic state (Fig. 4C and 4D). Conversely, the muscle regenerative capacity was severely compromised, as evidenced by the sharp decline in myogenic differentiation markers such as myogenin, MyoD, and MyHC (^###^*p* < 0.001 for all) relative to those in the healthy controls (Fig. 4E–4G).

Treatment with *L. fermentum* ANC4 modulated these molecular shifts, although the efficacy varied depending on the bacterial preparation. The live form (AH) was a more potent inhibitor of catabolic signaling, showing significant downregulation of GR (***p < 0.001) and Atrogin-1 expressions (***p < 0.001). In contrast, the heat-killed forms (HL and HH groups) did not show a statistically significant reduction in Atrogin-1 expression (Fig. 4D), suggesting a more limited impact on this specific proteolytic pathway. Interestingly, a divergent and specialized effect was observed in the restoration of myogenic and structural markers by the heat-killed preparations.

While the live groups significantly restored myogenin, MyoD, and MyHC levels, the heat-killed forms exhibited distinct advantages. Specifically, the HL group significantly increased myogenin expression (***p < 0.001, Fig. 4E), whereas the HH group elicited a statistically significant increase in MyoD expression (***p < 0.001, Fig. 4F). Most notably, both heat-killed groups exhibited a superior advantage in structural restoration, with HH showing a significantly more robust increase in MyHC expression compared to the AH group (^$$$^*p* < 0.001, Fig. 4G). These results suggest that live and heat-killed *L. fermentum* ANC4 offer complementary therapeutic benefits by targeting distinct aspects of muscle homeostasis, where the live form primarily inhibits proteolysis and the heat-killed form enhances myogenic and structural regeneration.

### *L. fermentum* ANC4 Induces Histological Improvements in Muscle Fiber Morphology and Fibrosis

**Fig. 5:**
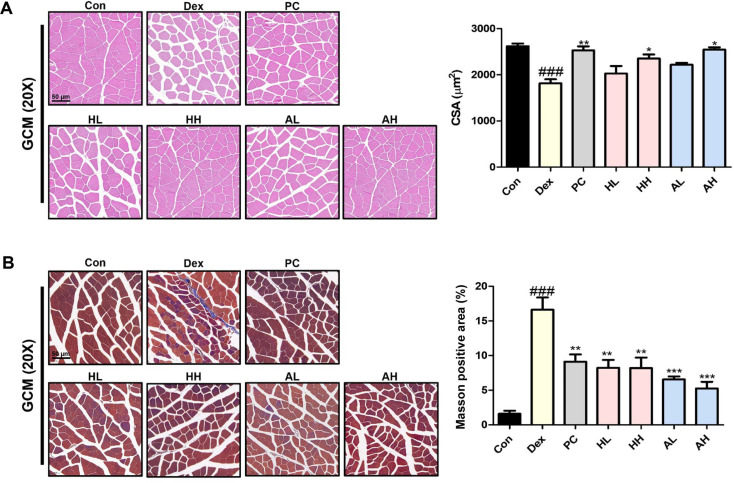
Histological analysis of muscle morphology and fibrosis in the gastrocnemius (GCM) muscle. (**A**) Hematoxylin and eosin (H&E) staining was used to assess the cross-sectional area (CSA) of muscle fibers. (**B**) Masson’s trichrome staining was performed to evaluate collagen deposition as a marker of muscle fibrosis. Quantitative image analysis for both CSA and the Masson positive area was performed using ImageJ software, applying standardized tracing and thresholding criteria to ensure reproducibility. ^###^*p* < 0.001 vs. control group (Con). *p < 0.05, **p < 0.01, and ***p < 0.001 vs. Dex-induced group (Dex). All data represent the mean ± SEM (n = 3–4 per group; five randomly selected fields per slide were evaluated).

To visualize the structural impact of atrophy, we stained the GCM muscle sections with H&E (Fig. 5A). As expected, the Dex-treated group showed significantly shrunken muscle fibers, with a CSA dropping to 1981.3 ± 87.17 μm^2^ (^###^*p* < 0.001). However, oral administration of *L. fermentum* ANC4 counteracted this reduction in a dose-dependent manner. The most robust recovery was observed in the high-dose groups; the heat-killed (HH) group recorded a CSA of 2352.94 ± 88.68 μm^2^ (*P < 0.05), whereas the live (AH) group reached 2547.45 ± 49.90 μm^2^ (*p < 0.05). These values were statistically comparable with those in the PC group (2529.67 ± 86.47 μm^2^, ***p < 0.001), indicating that both forms of *L. fermentum* ANC4 effectively protect muscle architecture against catabolic stress.

Next, we assessed fibrotic remodeling as a key marker of muscle quality degradation using Masson's trichrome staining (Fig. 5B). The Dex-induced group showed extensive collagen deposition, covering 16.60 ± 2.04% of the tissue area (^###^*p* < 0.001), which is a clear sign of fibrosis [21]. Conversely, *L. fermentum* ANC4 treatment drastically reduced pathological accumulation. We recorded fibrosis levels of 5.24 ± 1.11% for HL (*p < 0.01), 8.19 ± 1.53% for HH (**p < 0.01), 6.55 ± 0.43% for AL (**p < 0.001), and 5.24 ± 1.11% for AH (***p < 0.001). Notably, the AH group maintained fibrosis levels comparable with those in the PC group (9.12 ± 1.21%, **p < 0.01). These histological findings confirm that *L. fermentum* ANC4 maintains fiber size and actively suppresses fibrotic scarring, aligning well with the functional and molecular improvements observed in this study.

### *L. fermentum* ANC4 Restores MyoD Expression in GCM

**Fig. 6:**
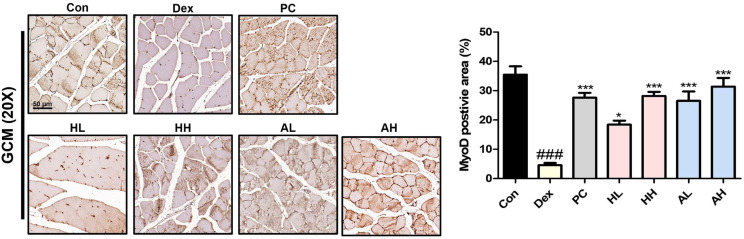
Immunohistochemical analysis of MyoD expression in the gastrocnemius (GCM) muscle. Representative immunohistochemical images of GCM tissues were analyzed to assess MyoD expression levels across treatment groups. Quantification was performed using ImageJ software. ^###^*p* < 0.001 vs. control group (Con). *p < 0.05 and ***p < 0.001 vs. Dex-induced group (Dex). All data represent the mean ± SEM (n = 3 per group; five randomly selected fields per slide were evaluated).

To spatially validate the regenerative changes observed in our western blot analysis, we performed immunohistochemical staining for MyoD in GCM tissue sections (Figs. 3 and 6). As expected, Dex-treated muscles displayed severe depletion of MyoD-positive nuclei, with the frequency dropping to 4.58 ± 0.73% (^###^*p* < 0.001). This visual scarcity confirmed that the intrinsic regenerative machinery of the muscle was deeply suppressed. However, treatment with *L. fermentum* ANC4 restored this machinery. We observed a significant and dose-dependent recovery in the number of MyoD-positive cells across the treatment groups. The MyoD-positive nuclei in the high-dose heat-killed (HH) group reached 28.17 ± 1.42% (***p < 0.001), whereas those in the live (AH) group reached 31.41 ± 2.94% (***p < 0.001). Notably, the AH group restored MyoD expression to levels comparable to or even slightly exceeding those in the positive control group (27.59 ± 1.67%, ***p < 0.001 vs Dex). This robust staining intensity provides compelling evidence that *L. fermentum* ANC4 actively stimulates muscle satellite cells, thereby reinforcing the myogenic signaling pathways essential for tissue repair.

### *L. fermentum* ANC4 Attenuates Dex-Induced Histological Alterations in the Liver and Kidneys

**Fig. 7:**
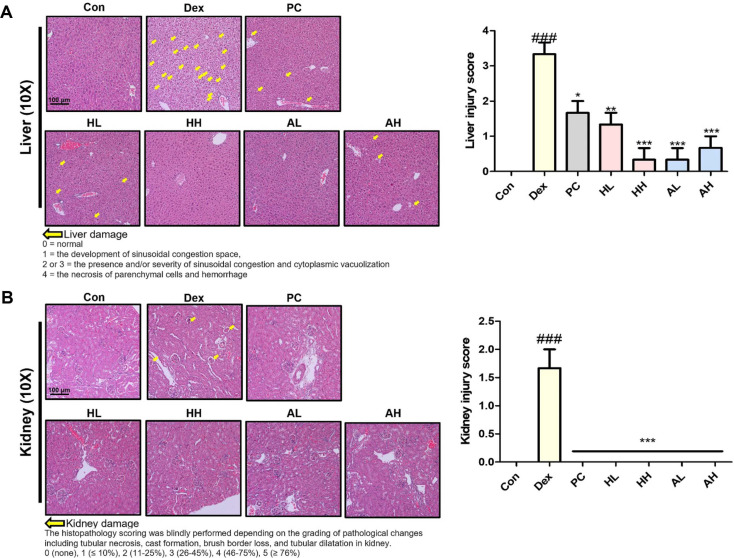
Histological evaluation of liver and kidney tissues following *L. fermentum* ANC4 administration in Dex-induced mice. Histological analysis of the liver and kidney performed using hematoxylin-eosin (H&E) staining. (**A**) Liver tissue was assessed using H&E staining, and injury scores were graded as follows: 0 = normal; 1 = sinusoidal congestion; 2–3 = increasing severity of congestion and cytoplasmic vacuolization; 4 = necrosis and hemorrhage of parenchymal cells. (**B**) Kidney histopathology was evaluated based on the extent of tubular necrosis, cast formation, brush border loss, and tubular dilatation, with scores defined as: 0 (none), 1 (≤ 10%), 2 (11–25%), 3 (26–45%), 4 (46–75%), and 5 (≥ 76%). ^###^*p* < 0.001 vs. control group (Con). *p < 0.05, **p< 0.01 and ***p < 0.001 vs. Dex-induced group (Dex). All data represent the mean ± SEM (n = 3 per group; five randomly selected fields per slide were evaluated).

To determine whether the detrimental effects of dexamethasone extended beyond the skeletal muscle, we histologically assessed the liver and kidneys using H&E staining (Fig. 7). Microscopic examination of liver tissue sections from the Dex-treated group revealed distinct signs of toxicity, characterized by pronounced sinusoidal congestion and cytoplasmic vacuolization. Consequently, the histopathological injury score increased to 3.33 ± 0.33 (^###^*p* < 0.001), indicating moderate-to-severe hepatic damage (Fig. 7A). However, the *L. fermentum* ANC4 intervention attenuated this effect in a dose-dependent manner. The high-dose groups demonstrated remarkable recovery; with the live form (AH), the histopathological injury score was reduced to 0.67 ± 0.33 (***p < 0.001), and the heat-killed form (HH) decreased it further to 0.33 ± 0.33 (***p < 0.001). These improvements were comparable to, or even numerically superior to those in the PC group (1.67 ± 0.33, *p < 0.05), suggesting potent hepatoprotective activity.

Similarly, the kidneys were not spared from Dex-induced structural damage. The Dex group showed severe pathological alterations, including tubular necrosis, cast formation, and tubular dilatation (Fig. 7B), resulting in an elevated injury score of 1.67 ± 0.33 (^###^*p* < 0.001). In contrast, *L. fermentum* ANC4 treatment effectively shielded the renal tissue from structural damage. Regardless of the form (live or heat-killed) or dose, all treatment groups (HL, HH, AL, and AH) showed significantly reduced renal injury scores compared with that of the Dex group (***p < 0.001), indicating preservation of normal renal morphology.

These findings confirm that *L. fermentum* ANC4 does not merely target muscle tissue but offers broad-spectrum systemic protection. By mitigating the degenerative responses in major metabolic organs, such as the liver and kidneys, *L. fermentum* ANC4 demonstrates potential in alleviating Dex-induced histological damage in metabolic organs such as the liver and kidneys.

## Discussion

In this study, we demonstrated that *L. fermentum* ANC4 effectively counteracts the muscle-wasting effects of Dex, with clear improvements in muscle function, mass, and histological integrity. These benefits appear to stem from three key actions: modulation of GR signaling, inhibition of ubiquitin-proteasome system (UPS)-mediated proteolysis, and stimulation of myogenic pathways. Thus, *L. fermentum* ANC4 emerges as a viable probiotic and postbiotic candidate for muscle preservation.

We first investigated the effect of *L. fermentum* ANC4 on catabolic processes that trigger atrophy. The UPS is the primary driver of protein breakdown during muscle atrophy [28]. Dex accelerates this process by binding to cytoplasmic GR, which then moves to the nucleus to activate E3 ubiquitin ligases, such as MuRF-1 and Atrogin-1 [29]. This signaling cascade amplifies UPS activity, leading to rapid protein degradation. Our results confirmed this mechanism as the Dex-treated group showed significantly elevated GR, MuRF-1, and Atrogin-1 levels (Fig. 4B–4D).

Although GR activation is fundamentally mediated by its translocation from the cytoplasm to the nucleus, the 2.5-folds increase in total GR protein levels observed in our Dex-treated group is consistent with established models of glucocorticoid-induced muscle atrophy, where increased receptor abundance correlates with enhanced catabolic signaling (Fig. 4B) [27]. Furthermore, the subsequent upregulation of downstream E3 ubiquitin ligases, MuRF-1 and Atrogin-1, provides functional evidence of GR-mediated transcriptional activity, as their expression levels are tightly coupled with the rate of glucocorticoid-induced proteolysis (Fig. 4C and 4D) [27, 30]. Future studies focusing on nuclear-cytoplasmic partitioning will further refine the precise inhibitory mechanism of *L. fermentum* ANC4 on GR translocation.

However, treatment with *L. fermentum* ANC4 successfully reversed these changes by downregulating both the upstream GR signaling and downstream proteolytic enzymes. This aligns with findings with other strains, such as *L. curvatus* CP2998, which also reduced UPS markers in Dex-treated models [31]. Interestingly, the live form of *L. fermentum* ANC4 suppressed Atrogin-1 more effectively compared with its heat-killed counterpart. The protective effects observed in this study suggest a potential involvement of the gut-muscle axis in mitigating Dex-induced atrophy. We hypothesize that the superior efficacy of live *L. fermentum* ANC4 in suppressing Atrogin-1 expression may be associated with its ability to modulate the intestinal environment. Although not directly measured in this study, previous reports have indicated that live probiotic strains can promote the production of short chain fatty acids (SCFAs) such as butyrate, which are known to influence systemic inflammation and muscle protein metabolism [32-34]. Therefore, it is speculative but plausible that the continuous administration of live ANC4 provided a more consistent supply of such beneficial metabolites compared to the heat killed form. However, further investigations including gut microbiome profiling and fecal SCFA measurements are essential to definitively confirm these metabolic pathways. Specifically, butyrate inhibits FOXO3a as a key activator of MuRF-1 and Atrogin-1, thereby limiting proteolysis and preventing muscle dysfunction [35, 36].

In addition to suppressing catabolism, *L. fermentum* ANC4 treatment significantly enhanced myogenic regenerative responses. We observed increased expression of key myogenic markers, including MyoD, myogenin, and MyHC, indicating active muscle regeneration (Fig. 4E–4G). Interestingly, while the positive control (Oxymetholone) group showed clear improvements in early myogenic markers, its MyHC levels did not show a statistically significant increase. This may be attributed to the 14-day experimental duration, which might have been sufficient for the initial activation of myogenic machinery and cellular swelling but insufficient for the full synthesis and stable accumulation of large structural proteins like MyHC. Future studies with extended administration periods may be required to observe the complete structural recovery induced by pharmacological anabolic agents.

This is consistent with previous reports on other *L. plantarum* strains; for instance, *L. plantarum* HY7715 promoted myogenesis in a sarcopenia model [37], whereas *L. plantarum* WJL enhanced IGF-1 expression to counter malnutrition-induced wasting [38]. Conversely, the heat-killed form of *L. fermentum* ANC4 elicited a stronger upregulation of MyHC than did the live form. This distinct response suggests that structural components of the bacterial cell wall, such as peptidoglycans or lipoteichoic acids, may interact with host pattern recognition receptors (*e.g.*, Toll-like receptors) to trigger immune-mediated regenerative signals [35, 37]. Our immunohistochemical analysis supported this finding, showing restoration of MyoD-positive nuclei that had been depleted by Dex. This confirms that *L. fermentum* ANC4 contributes to recovery by reactivating the intrinsic regenerative machinery of the muscles.

Furthermore, a critical aspect of Dex-induced muscle atrophy is its impact on specific muscle groups, particularly those like the GCM, which is predominantly composed of fast twitch (Type II) fibers [39]. In this study, we observed that the protective effects of *L. fermentum* ANC4 were most pronounced in the GCM muscle. Given that the GCM is predominantly composed of fast-twitch fibers, its mass and functional performance (grip strength and treadmill endurance) were significantly preserved. Although these results demonstrate the overall efficacy of *L. fermentum* ANC4 in shielding GCM architecture from dexamethasone induced proteolysis, we did not perform direct fiber type differentiation experiments, such as IHC staining for Type IIa, IIb, or IIx markers. Therefore, while our findings suggest a strong protective role in fast twitch dominant muscles, further studies employing specific fiber type markers are necessary to definitively elucidate whether *L. fermentum* ANC4 exerts a truly fiber type specific targeted efficacy.

Morphological assessment using H&E staining further validated the muscle-protective properties of *L. fermentum* ANC4 (Fig. 5). Although Dex treatment caused the expected shrinkage in muscle fiber CSA, both forms of *L. fermentum* ANC4 restored fiber size in a dose-dependent manner (Fig. 5A). A particularly significant finding of our study was the reduction in fibrosis. Masson's trichrome staining revealed that *L. fermentum* ANC4 treatment significantly reduced collagen deposition. Although muscle mass preservation by *Lactobacillus* strains is well-documented [39-41], evidence regarding their impact on fibrosis remains scarce. Therefore, the observed antifibrotic effect represents a novel and therapeutically relevant finding for managing glucocorticoid-induced muscle damage. This suggests that *L. fermentum* ANC4 helps maintain muscle quality and prevents fibrotic remodeling, which often leads to functional decline.

The observed structural benefits directly translate into functional improvements. Mice treated with *L. fermentum* ANC4 showed a significant recovery in both grip strength and treadmill endurance (Fig. 2). These results indicate that strain does more than just maintain muscle size; it effectively mitigates the functional deficits caused by glucocorticoids. We attribute this recovery to the combined effects of restored MyoD expression and maintenance of fiber CSA. Finally, although we primarily focused on biological activity, we verified the safety profile of this strain. Beyond its local effects on the muscle, *L. fermentum* ANC4 alleviated histopathological damage in the liver and kidneys, indicating its potential to reduce Dex-induced histopathological alterations in major organs. Furthermore, *L. fermentum* ANC4, isolated from traditional *makgeolli*, demonstrated antimicrobial susceptibility consistent with EFSA standards (Table S1), supporting its potential for safe clinical and commercial applications.

Despite the significant findings of this study, several limitations should be acknowledged. First, the high-dose Dex-induced atrophy model used here represents an acute pharmacological state of muscle wasting. Therefore, our observations regarding the efficacy of *L. fermentum* ANC4 may be primarily restricted to glucocorticoid-induced atrophy and may not fully recapitulate the complex pathophysiological features of aging-related sarcopenia or cancer-induced cachexia. Furthermore, a limitation of the present study is the absence of direct evidence for gut–muscle axis modulation, such as gut microbiota profiling and intestinal permeability markers. While our data clearly demonstrate the anti-atrophic effects of *L. fermentum* ANC4 within the skeletal muscle tissue, the precise mediation through the gut–muscle axis remains a plausible hypothesis rather than an experimentally demonstrated mechanism. Future investigations using specialized aging models and advanced microbiome profiling are necessary to validate these interactions and expand the therapeutic implications of *L. fermentum* ANC4.

The exclusive utilization of male mice represents a primary limitation of the current study. Although this experimental strategy was implemented to ensure physiological homogeneity and minimize confounding variables associated with the estrus cycle, existing literature underscores the significance of sex specific dimorphism in glucocorticoid sensitivity and skeletal muscle composition. Consequently, the anti-atrophic efficacy of *L. fermentum* ANC4 requires further validation in both male and female cohorts to ensure the broad generalizability and clinical relevance of these findings across diverse biological populations.

## Conclusion

In summary, our study confirms that *L. fermentum* ANC4 can effectively combat the muscle-wasting effects of Dex. This protection is achieved through a multifaceted mechanism that dampens glucocorticoid receptor signaling, blocks UPS-mediated protein breakdown, and actively stimulates muscle regeneration. Our findings provide novel insights by demonstrating that live and heat-killed forms of *L. fermentum* ANC4 offer distinct yet complementary benefits; live cells primarily inhibit proteolysis, whereas heat killed cells are significantly more effective at promoting MyHC structural regeneration. Furthermore, we documented a novel anti-fibrotic efficacy and fast twitch targeted protection, which distinguish *L. fermentum* ANC4 as a versatile candidate for preserving muscle structure and function while mitigating structural damage in the liver and kidneys. These results position *L. fermentum* ANC4 as a promising probiotic or postbiotic candidate. However, as these findings are based on an acute high-dose pharmacological model, they should be interpreted with caution regarding their direct clinical application. Moving forward, the efficacy of this strain needs to be validated in chronic or age-related clinical settings to further investigate the potential involvement of the gut muscle axis interactions using advanced microbiome and metabolomic profiling.

## Supplemental Materials

Supplementary data for this paper are available on-line only at http://jmb.or.kr.
