# Supplementary material for: Limosilactobacillus fermentum ANC4 (KCTC 15072BP) Mitigates Dexamethasone-Induced Muscle Atrophy and Improves Overall Skeletal Muscle Function
Source: J Microbiol Biotechnol. 2026 Apr 28;36:e2603025. doi: 10.4014/jmb.2603.03025 (PMC13146494; doi:10.4014/jmb.2603.03025)
Supplement: Supplementary file 1 [file jmb-36-e2603025-supple.pdf]

J. Microbiol. Biotechnol. 2026. 36: e2603025

https://doi.org/10.4014/jmb.2603.03025

Supplementary Table and Figure

Table S1. Minimum inhibitory concentration (MIC) of *L. fermentum* ANC4 compared with EFSA antimicrobial cut-off values

Antibiotics	*L. fermentum* ANC4 MIC (μg/mL)	EFSA cut-off (μg/mL)
Ampicillin	< 0.125	2
Gentamycin	8	16
Kanamycin	32	64
Streptomycin	32	64
Erythromycin	< 0.125	1
Clindamycin	< 0.125	4
Tetracycline	4	8
Chloramphenicol	4	4

Fig. 4.

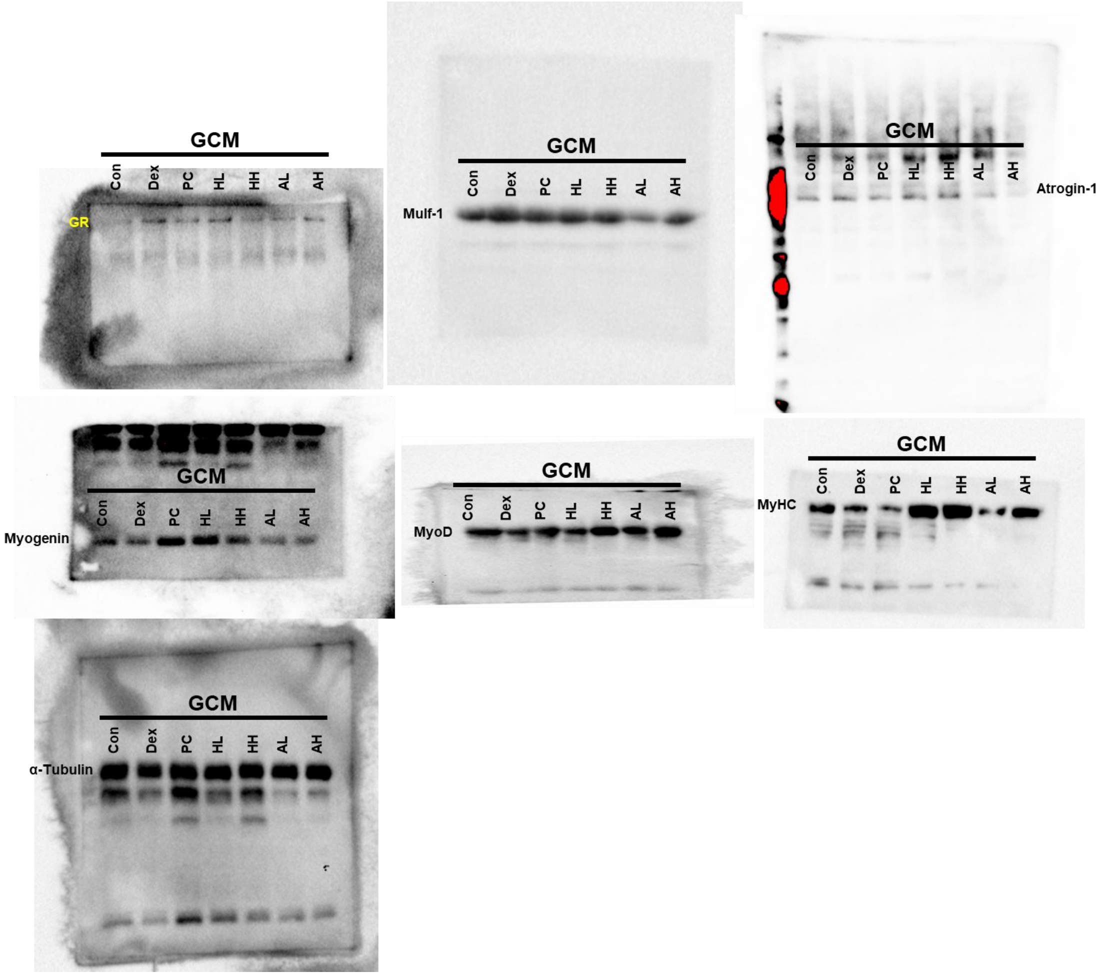

<Western blotting assay_raw images>
